# Evaluation of eligibility criteria in living donor liver transplantation for hepatocellular carcinoma by α-SMA-positive cancer-associated fibroblasts

**DOI:** 10.3892/or.2013.2616

**Published:** 2013-07-16

**Authors:** HIROYUKI TAKAMURA, SHINICHI NAKANUMA, HIRONORI HAYASHI, HIDEHIRO TAJIMA, KAHEITA KAKINOKI, SEISYO SAKAI, ISAMU MAKINO, HISATOSHI NAKAGAWARA, TOMOHARU MIYASHITA, KOICHI OKAMOTO, KEISHI NAKAMURA, KATUNOBU OYAMA, MASASHI INOKUCHI, ITASU NINOMIYA, HIROHISA KITAGAWA, SACHIO FUSHIDA, TAKASHI FUJIMURA, ICHIRO OHNISHI, MASATO KAYAHARA, TAKASHI TANI, KUNIAKI ARAI, TARO YAMASHITA, TATSUYA YAMASHITA, HOSHIKO KITAMURA, HIROKO IKEDA, SHUICHI KANEKO, YASUNI NAKANUMA, OSAMU MATSUI, TETSUO OHTA

**Affiliations:** 1 Department of Gastroenterologic Surgery, Graduate School of Medicine, Kanazawa University, Kanazawa, Ishikawa 920-8641, Japan; 2 Department of Gastroenterology, Graduate School of Medicine, Kanazawa University, Kanazawa, Ishikawa 920-8641, Japan; 3 Department of Diagnostic Pathology, Graduate School of Medicine, Kanazawa University, Kanazawa, Ishikawa 920-8641, Japan; 4 Department of Human Pathology, Graduate School of Medicine, Kanazawa University, Kanazawa, Ishikawa 920-8641, Japan; 5 Department of Radiology, Graduate School of Medicine, Kanazawa University, Kanazawa, Ishikawa 920-8641, Japan

**Keywords:** living donor liver transplantation, hepatocellular carcinoma, cancer-associated fibroblast, Up-to-seven criteria, α-smooth muscle actin

## Abstract

The eligibility criteria of liver transplantation (LT) for hepatocellular carcinoma (HCC) must clearly confirm the prognosis not only from pathological diagnosis but also from pre-operative imaging diagnosis. In the present study, we evaluated published eligibility criteria for LT based on both pre-operative imaging diagnosis and pathological diagnosis using living donor liver transplantation (LDLT) recipients at our hospital by α-smooth muscle actin (SMA)-positive cancer-associated fibroblasts (CAFs) in HCC. The Up-to-seven (Up-to-7), Asan and Tokyo criteria were evaluated, in both overall survival and HCC disease-free survival, to be statistically significantly beneficial criteria to define post-LDLT prognosis. Recipients only within Up-to-7 criteria based on both pre-operative imaging diagnosis and pathological diagnosis survived without HCC recurrence. Recipients with proliferation of α-SMA-positive CAFs in HCC had significantly poorer prognosis. All survival recipients without HCC recurrence, who were above the Up-to-7 criteria in pathological diagnosis, had no proliferation of α-SMA-positive CAFs. As a result of multivariate analysis, the significant independent factors defining prognosis of recipients after LDLT for HCC were Up-to-7 criteria and proliferation of α-SMA-positive CAFs. The ideal eligibility criteria for LDLT with HCC is Up-to-7 criteria and α-SMA-positive CAFs was considered to be an important factor in HCC recurrence. LDLT should be limited to recipients within Up-to-7 criteria or without proliferation of α-SMA-positive CAFs.

## Introduction

In 1996, eligibility criteria such as the Milan criteria (MC) of liver transplantation (LT) for hepatocellular carcinoma (HCC) were reported by Mazzaferro *et al*(1). MC emphasized LT as a therapeutic option for patients with HCC. Living donor liver transplantation (LDLT) is virtually the only option for patients with HCC in the east Asian countries such as Korea (2) or Japan (3–9), where the number of deceased donors is limited, and for patients above MC in western countries such as the United States and in Europe. Therefore, understanding how far the criteria of LT for HCC can be extended in LDLT from MC is key in improving the outcomes in regions with limited organ donors. There have been several reports of expanded criteria as indications of LT for HCC, such as the Up-to-seven (Up-to-7) criteria (10), University California of San Francisco (UCSF) criteria (11), Asan criteria (2), Tokyo (5–5 rule) criteria (3), Kyoto criteria (4,5) and Kyushu criteria (6–8). In addition, Kyoto criteria (4,5) and Kyushu criteria (6–8) showed pre-operative tumor markers such as the des-γ-carboxyprothrombin (DCP) level. In the present study, we evaluated the predictive values of the previously proposed selection criteria, including Up-to 7 criteria, UCSF criteria, Asan criteria, Tokyo criteria, Kyoto criteria and Kyushu criteria, on the overall survival (OS) and HCC disease-free survival (DFS) of LDLT recipients with HCC. These criteria are categorized into several types which are based only on pre-operative imaging diagnosis, or on pathological diagnosis of the explant liver, which consider microvascular invasion as above criteria, and take account of tumor markers. According to Japanese national data (9), in addition to the MC, it is reported that the values of tumor markers [α-fetoprotein (AFP) and DCP] define prognosis, but as various factors are involved in tumor markers, it is difficult to incorporate them into international eligibility criteria of LT for HCC. Regardless of whether it is deceased donor liver transplantation (DDLT) or LDLT, the criteria of LT for HCC should be defined solely by simple factors such as tumor diameter or number to guarantee their international applicability. Furthermore, eligibility criteria of LT for HCC must significantly define the prognosis for recipients in evaluations which are based not only on pathological diagnosis of the explant liver, but also on pre-operative imaging diagnosis. However, it is important to perform pre-operative imaging diagnosis of HCC close to post-operative pathological diagnosis. If the accuracy of imaging diagnosis of HCC is low, the reliability of the criteria decreases, therefore, pre-operative imaging diagnosis should be performed accurately using some imaging diagnostic modalities. In order to enhance imaging diagnostic accuracy for HCC, in addition to dynamic multi-detectable-row computer tomography (dynamic MDCT) and gadolinium ethoxybenzyl diethylenetriamine pentaacetic acid-enhanced magnetic resonance imaging (Gd-EOB-DTPA-MRI) (12,13), we also obtained images as far as possible using CT under angiography [during arterial portography (CTAP) and during hepatic arteriography (CTHA)] (14–17). In view of the fact that a healthy living donor is exposed to major risks by hepatectomy, recurrence of HCC after LT in the recipient must be avoided. To receive LDLT under Japanese health insurance, although no restrictions are imposed as to therapeutic history 3 months prior to LT, the recipient must satisfy the MC in the pre-operative final imaging diagnosis. Eligibility criteria have been reported by various high-volume centers in Japan (3–8) and there are attempts to widen eligibility of LDLT for HCC under health insurance. In this context, in order to expand the eligibility criteria from within MC, we evaluated which criteria were the most suitable from the two viewpoints of pre-operative imaging diagnosis and pathological diagnosis with recipients who had performed precise pre-operative diagnostic imaging and had been observed for >5 years after LDLT for HCC. Furthermore, we evaluated the appropriateness of the above criteria from the viewpoint of proliferation of α-smooth muscle actin (SMA)-positive cancer-associated fibroblasts (CAFs), which are strongly related to cancer progression and invasion (18,19). However, there are no reports which evaluate HCC recurrence after LT from the viewpoint of α-SMA-positive CAFs. We therefore evaluated the relationship between HCC recurrence after LDLT and proliferation of α-SMA-positive CAFs, as well as the correlation between eligibility criteria and α-SMA-positive CAFs.

## Materials and methods

### Patients

From July 2003 to December 2007, 22 consecutive LDLTs for liver cirrhosis (LC) with HCC were performed at Kanazawa University Hospital (Ishikawa, Japan) after receiving approval from the Ethics and Indications Committee of Kanazawa University. Our selection criteria for the patients with HCC were as follows: no modality except LDLT available to cure patients with HCC and end-stage liver disease, no extrahepatic metastasis and no macrovascular invasion such as portal vein or hepatic vein infiltrations. We limited adaptation of LDLT for HCC to within MC under health insurance of Japan since January 2008, but performed LDLT for above MC recipients by own expenses until December 2007. Therefore targeted cases for the present study were limited to recipients who had undergone LDLT by December 2007. Twenty-two patients had HCC, proven histologically. The median age of the 22 patients was 55.5 years (range, 47–64 years). Written informed consent for the present study was obtained from each patient. In addition, the study was approved by the Kanazawa University Ethics Committee. Tumor-specific evaluations, including abdominal and thoracic dynamic MDCT, abdominal CTAP, abdominal CTHA, abdominal Gd-EOB-DTPA-MRI, bone scintigraphy, and the determination of AFP and DCP (Protein induced by Vitamin K, PIVKA-II), were performed for all LDLT candidates. The diameter and number of HCCs were determined by multiple radiologists, based on pre-operative imaging studies within one month of LT. Thus, the variables used in the criteria, including tumor diameter and number, were based on these data. The explants were examined histologically. For pathological examination, whole liver explants were fixed in 10% formalin and cut into 5-mm slices to facilitate gross and histological examinations. Following macroscopic examination, the nodular lesions were embedded in paraffin, cut into 4-inch sections and stained with hematoxylin and eosin. The incidence of microvascular invasion and histological grades were subsequently estimated within these criteria. Microvascular invasion was defined as microscopic portal vein or hepatic vein invasion of cancer cells. The stage was determined for each patient according to the AJCC/UICC (6th edition) guidelines (20) and UNOS TMN (21). Among the 22 patients, 10 (45.5%) met the MC according to pre-operative first imaging diagnosis, while 12 did not. According to previous studies, Up-to 7, UCSF, Asan, Tokyo, Kyoto and Kyushu criteria were applied and the predictive impacts of these criteria for HCC recurrence were evaluated by univariate analyses. The previously proposed selection criteria for HCC are briefly described below and are shown in Table I. The Up-to-7 criteria are defined as HCC with seven as the sum of the diameter of the largest tumor (in cm) and the number of tumors. The UCSF criteria are defined as HCC meeting the following criteria: solitary tumor of ≤6.5 cm, or ≤3 nodules with the largest lesion of ≤4.5 cm and a total tumor diameter of ≤8 cm. The Asan criteria are defined as HCC meeting the following criteria: tumor up to 6 nodules with a maximum diameter of 5 cm without gross vascular invasion. The Tokyo criteria are defined as HCC meeting the following criteria: tumor of up to 5 nodules with a maximum diameter of 5 cm (5–5 rule) that are evaluated with pre-operative imaging. The Kyoto criteria are defined as HCC meeting the following criteria: ≤10 tumors that are all ≤5 cm in diameter and DCP of ≤400 mAU/ml. The Kyushu University criteria are defined as HCC with tumor diameter <5 cm or DCP <300 mAU/ml. In the 7 above MC recipients who underwent pre-LDLT therapy to downstage HCC, transarterial chemo-lipiodolisation (TACL) was performed in all cases, and radiofrequency ablation therapy (RFA) was also performed in 2 cases. The recipients who underwent pre-LDLT therapy for HCC were observed for ≥3 months from the end of the pre-operative therapy to LDLT. There were 4 out of 5 recipients who were downstaged from above MC (pre-operative first imaging diagnosis) to within MC (pre-operative final imaging diagnosis) by pre-LDLT therapy. In the 15 cases were LDLT was performed without prior therapy, the pre-operative first imaging diagnosis was considered the pre-operative final imaging diagnosis. There were 13 cases in total that received therapy for HCC in the past before LDLT; TACL had been performed in 11 cases and several treatments in 10 cases. RFA had been performed in 8 cases, percutaneous ethanol injection therapy (PEIT) in 5 cases and hepatectomy in 2 cases. In addition, transarterial infusion chemotherapy had been performed in only 1 case. The clinical follow-up of patients after LDLT for HCC followed a strict protocol, which did not change during the study period. The patients were seen biweekly for the first 6 months and then monthly. The patients underwent enhanced MDCT or Gd-EOB-DTPA-MRI at 4–6 month intervals. Liver biopsy, hepatic angiography with CT, bone scintigraphy or 2-Fluoro 2-deoxyglucose positron emission tomography (FDG-PET) CT was also performed if deterioration in the graft function or a rise in the AFP or DCP levels was noted. The mean follow-up period was 7 years.

### Immunohistochemistry

The proliferation of α-SMA-positive CAFs was evaluated immunohistologically. When several tumors were present, the tumor with microvascular invasion was evaluated. If no microvascular invasion was found, tumors which had the poorer histological degree of differentiation or differentiated into biliary tract type (CK7-positive or CK19-positive), were evaluated. Tumor specimens were fixed in 10% formalin and embedded in paraffin. The expressions of α-SMA in HCC were examined immunohistochemically using respective primary antibodies using EnVision^+^ System (DAKO). De-waxed 4-μm sections were incubated with 1:50 with protein blocking serum for 10 min to block non-specific binding and immunostaining was performed using EnVision^+^ System. Briefly, the slides were incubated with each primary antibody (1:50) at 4°C overnight. After washing, the EnVision^+^ polymer solution was applied for 1 h. The reaction products were visualized via a diaminobenzidine (DAB) reaction. The specimens were then lightly counterstained with hematoxylin and examined under a fluorescence microscope. Primary antibody used for immunostaining was Actin α2 Smooth Muscle rabbit anti-human polyclonal antibody (Novus Biologicals, Littleton, CO, USA).

### Computer-assisted image analysis (19)

We used computer-assisted image analysis to quantify the value of α-SMA expression in HCC. After staining for α-SMA, the histological sections were observed using a microscope equipped with a charge coupled-device color camera (Olympus Co., Japan) under constant electrical and optical conditions. A random selection of 10 fields in most poorly differentiated and α-SMA-positive CAF proliferating lesions of HCC were assessed for α-SMA expression. Using an imaging processor (VH Analyzer; Keyence Co., Japan) the percentage of α-SMA expression stromal area was quantified as the relative percentage of the α-SMA-positive stromal area to the selected fields of cancer.

### Statistical analysis

All statistical analyses were performed using SPSS Software v20 (IBM-Japan, Tokyo, Japan). The continuous variables were compared using the Mann-Whitney U test. All variables are expressed as means ± standard deviation (SD). The categorical data were compared using χ^2^ tests. We compared Kaplan-Meier distributions of time to mortality or HCC recurrence after LDLT with the log-rank test or generalized Wilcoxon. Cox’s proportional hazard model was used to identify independent variables for post-operative recurrence of HCC. The comparative evaluation was performed among the Milan, Up-to-7, Asan, Tokyo, Kyoto, Kyushu criteria, degree of α-SMA-positive CAFs and clinicopathological variables including pre-operative serum AFP levels and serum DPC levels, presence of microvascular invasion, histological grade of the tumor (poorly differentiated), the number of tumors and maximum diameter of tumor on the resected specimen. The differences were considered statistically significant when the P-value was <0.05.

## Results

Table II shows background characteristics of 22 recipients who underwent LDLT for HCC according to post-LDLT with or without HCC recurrence. The average age of recipients was 56 years, 17 of whom were males, and the average MELD score of recipients was 14 points. There were 12 recipients with hepatitis C viral (HCV) hepatitis and 10 recipients with hepatitis B viral (HBV) hepatitis. Seventeen recipients were given right hepatic graft and 5 recipients were given left hepatic graft with caudate lobe. The average graft volume/standard liver volume ratio (GV/SLV) (22) of recipients was 46%. The average age of the donors was 36 years. There were 5 cases with acute cellular rejection (ACR) after LDLT (23%). No operation-related mortality of recipients occurred. All donors returned to society promptly after the donor operation. For immunosuppressive drugs, tacrolimus (FK) was used in 17 cases (77.3%) and cyclosporine (CyA) was used in 5 cases. Administration of steroids (prednisone) was limited to 1 week after LDLT in 11 cases (50%), while in the remaining 11 cases administration was continued for a longer period of ≥6 months post-operatively. Mycophenolate mofetil (MMF) was also used in 13 cases (59%). As to UNOS TNM, 2 cases were stage I, 4 cases were stage II and 16 cases (73%) were stage IV. Regarding UICC TNM, 2 cases were stage I, 18 cases (82%) were stage II and 2 cases were stage III. Concerning histological differentiation of HCC, well-differentiated HCC was only one case, moderately differentiated HCC were 15 cases (68%) which accounted for the majority, poorly differentiated HCC were 3 cases and the combined type were 3 cases. In the statistical study, well and moderately differentiated were both considered as differentiated type, while poorly and combined were both considered as poorly differentiated type. There were 13 recipients (59%) who had received prior therapy for HCC and 7 recipients who had received pre-LT therapy to downstage HCC. A total of 15 cases (68%) had a history of prior therapy or had received pre-operative therapy for HCC prior to LDLT. In the 7 cases where pre-LT therapy was performed to downstage HCC, 4 cases were downstaged from above MC to within MC. However, in 2 of these 4 cases where downstaging of HCC was attempted, recurrence of HCC was found after LT. To date, 8 recipients have died. The cause of mortality was HCC recurrence in 6 cases, accounting for the majority, liver failure due to recurrence of HCV hepatitis in 1 case and cancer in another organ (oropharyngeal carcinoma) in 1 case, but in all 8 cases, recurrence of HCC was found. HCC recurrence in post-LT recipients was found most often in graft liver, but lung metastasis, bone metastasis, adrenal metastasis and peritoneal dissemination or lymph node metastasis were also observed concurrently. There were no operation-related deaths in the recipients. As shown in Table II, a significant correlation of HCC recurrence after LT was found only with UNOS TNM.

**Figure 1:**
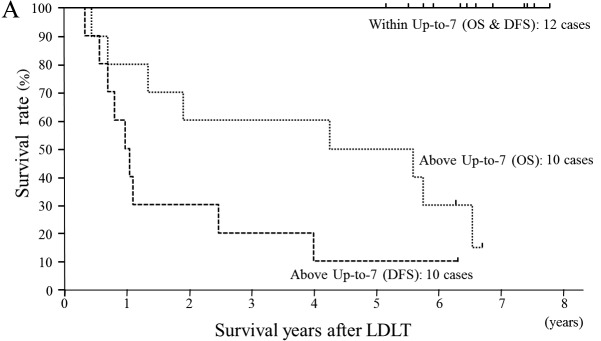

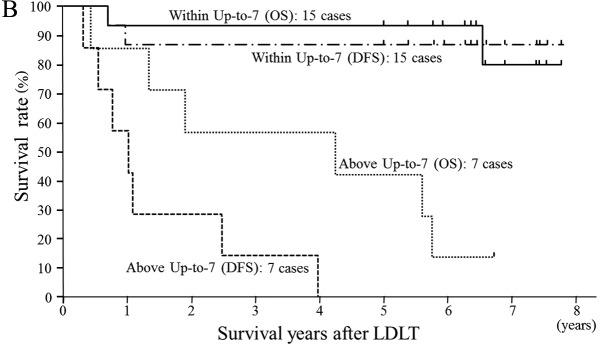

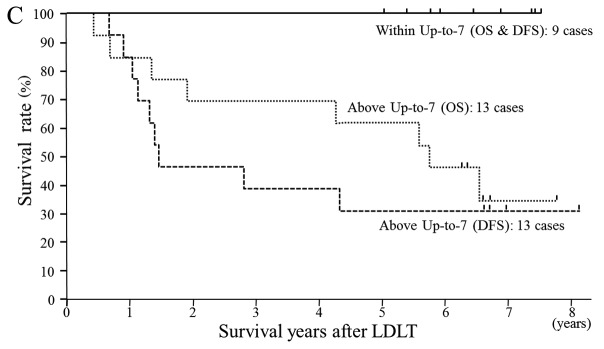
(A) Overall survival (OS) and hepatocellular carcinoma (HCC) disease-free survival (DFS) in living donor liver transplantation (LDLT) patients with HCC according to Up-to-seven (Up-to-7) criteria (permitting microvascular invasion) which were determined by pre-operative first imaging diagnosis. All 12 cases within Up-to-7 criteria survived without HCC recurrence. The OS and the DFS survival rates of within Up-to-7 criteria are statistically significantly (P<0.001) better than above Up-to-7 criteria. (B) OS and DFS in LDLT patients with HCC according to Up-to-7 criteria (permitting microvascular invasion) which were determined by final imaging diagnosis. There were only 2 recurrence cases in 18 cases within Up-to-7 criteria. The OS and the DFS of within Up-to-7 criteria are statistically significantly (P<0.001) better than above Up-to-7 criteria. (C) OS and DFS in LDLT patients with HCC according to Up-to-7 criteria (permitting microvascular invasion) which were determined by pathological diagnosis. All 9 cases within Up-to-7 criteria survived without HCC recurrence. The OS and the DFS of within the Up-to-7 criteria are statistically significantly (P<0.05) better than above the Up-to-7 criteria.

As shown in Fig. 1A and C, all cases judged as within Up-to-7 criteria in the pre-operative first imaging diagnosis or pathological diagnosis survived without HCC recurrence. On the other hand, in cases judged as above Up-to-7 criteria in the pre-operative first imaging diagnosis and pathological diagnosis, the 5-year DFS was 10 and 21%, respectively, which is poor prognosis. Fig. 1B shows the OS and DFS of recipients based on the Up-to-7 criteria evaluated by pre-operative final imaging diagnosis. Comparing OS and DFS of within Up-to-7 criteria with above criteria, a significant difference was found, and there were 2 cases of recurrence in within Up-to-7 criteria.

Table III shows the OS and DFS of recipients according to the MC, Up-to-7, Asan, Tokyo, Kyoto and Kyushu criteria based on the pre-operative first imaging diagnosis and pathological diagnosis. Recipients within Asan criteria which permit wider eligibility than Up-to-7 or UCSF criteria had a significantly better prognosis than above criteria, but there were 2 cases of HCC recurrence among the within criteria in pre-operative first imaging diagnosis. The Tokyo criteria gave the same results as the Asan criteria. According to the Asan criteria or Tokyo criteria, two deaths due to HCC recurrence were confirmed based on the pre-operative first imaging diagnosis, thus regarding eligibility for LDLT, the Up-to-7 criteria were deemed the most appropriate criteria. On the other hand, according to the Kyoto criteria, in an evaluation based on pre-operative final imaging diagnosis, there was a significant correlation with the prognosis of recipients, but in an evaluation based on the pathological diagnosis, the prognosis was not reflected. According to the Kyushu criteria, a significant difference was found in DFS between within criteria and above criteria, but for OS, there was no significant difference. These two criteria appear useful to distinguish patients at very high risk of HCC recurrence in a single high-volume center, but in the current situation where it is not possible to prevent recurrence and no particularly effective therapy after HCC recurrence, they cannot be considered universal standard criteria. The most appropriate criteria which define the prognosis of recipients after LDLT for HCC, for both pre-operative imaging diagnosis and pathological diagnosis, are the Up-to-7 criteria, and in view of the burden of living donors, it should be made the global standard of eligibility criteria for LT in HCC.

As shown in Table IV, the degree of histological differentiation of HCC, the values of serum AFP and serum DCP, and the presence of microvascular invasion were not significantly correlated with the prognosis after LDLT. In other words, microvascular invasion should be admitted as within criteria.

Proliferation of α-SMA-positive CAFs which is thought to be strongly related to cancer progression and invasion, clearly specifies the prognosis after LDLT in HCC. However, α-SMA was not found to be expressed in HCC cancer cells. We categorized the proliferation of α-SMA-positive CAF into the following 3 groups.

**Figure 2:**
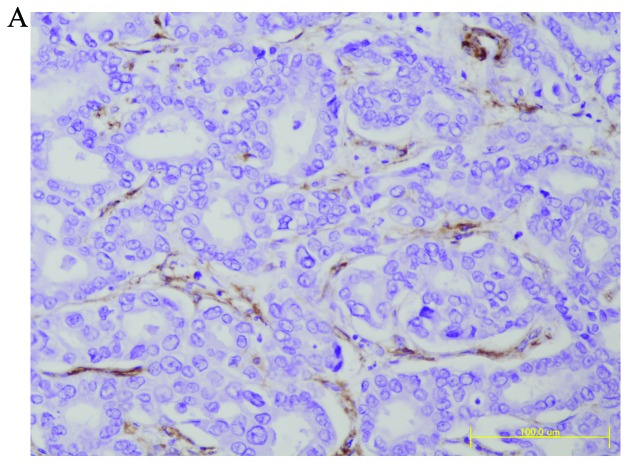

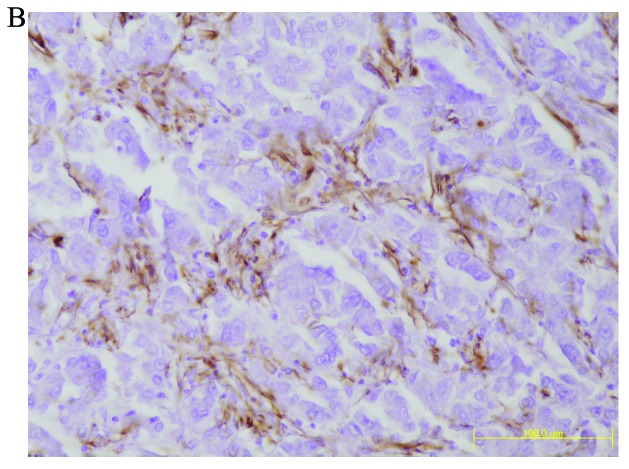

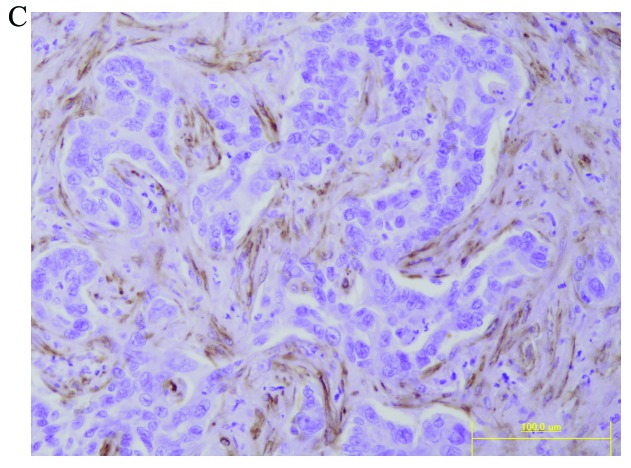
Representative immunohistochemical staining for α-smooth muscle actin (α-SMA) in hepatocellular carcinoma (HCC) tissue sections of living donor liver transplantation (LDLT) patients. (A) α-SMA positivity in cancer-associated fibroblasts (CAFs) of HCC is low grade (<1.0%). (B) α-SMA positivity in CAF of HCC is middle grade (<10%). (C) α-SMA positivity in CAF of HCC is high grade (≥10%).

Group I (Fig. 2A), 10 cases: low grade proliferation of α-SMA-positive CAFs; proliferation of cancer stroma not found, only slight proliferation of α-SMA-positive CAFs and staining was <1% of ten fields under high power view.

Group II (Fig. 2B), 8 cases: middle grade proliferation of α-SMA-positive CAFs; cancer nests were bordered over their whole circumference by α-SMA-positive CAFs, but the cancer stroma (CAFs) accounted for <10% of ten fields under high power view.

Group III (Fig. 2C), 4 cases: high grade proliferation of α-SMA-positive CAFs group; extensive proliferation of α-SMA-positive CAFs and cancer stroma accounted for >10% of ten fields under high power view.

**Figure 3:**
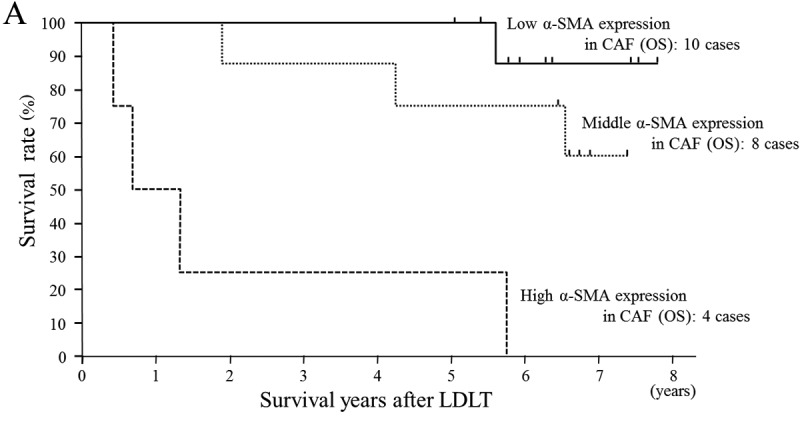

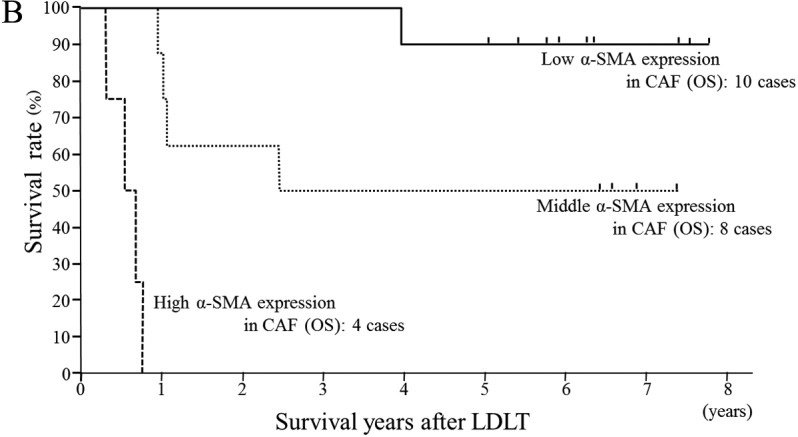
(A) Overall survival (OS) in living donor liver transplantation (LDLT) patients with hepatocellular carcinoma (HCC) according to proliferation of α-smooth muscle actin (α-SMA)-positive cancer-associated fibroblast (CAF) which was determined immunohistologically. Only one HCC recurrence patient died in 8 cases of low grade α-SMA-positive CAF group. All 4 cases of high grade α-SMA-positive CAF died due to HCC recurrence. The high grade α-SMA-positive CAF group had statistically significantly (P<0.05) poorer survival rates than the low and middle α-SMA-positive CAF groups. (B) Disease-free survival (DFS) in LDLT patients with HCC according to proliferation of α-SMA-positive CAF which was determined immunohistologically. There was only one HCC recurrence case in 8 cases of low grade α-SMA-positive CAF. All 4 cases of high grade α-SMA-positive CAF presented HCC recurrence soon after LDLT. The high grade α-SMA-positive CAF group had statistically significantly (P<0.01) poorer survival rates than the low and middle α-SMA-positive CAF groups, and middle grade α-SMA-positive CAF group had statistically significantly (P<0.05) poorer survival rates than the low grade group. There is a statistically significant correlation between post-LDLT HCC recurrence and α-SMA-positive CAF, i.e., α-SMA-positive CAF in HCC may be correlated with malignant potential of HCC progression and metastasis.

As shown in Fig. 3, as the proliferation of α-SMA-positive CAFs increased, recurrence of HCC increased significantly, and all 4 patients in group III died soon after LDLT due to recurrence of HCC. In above Up-to-7 criteria recipients, significant proliferation of α-SMA-positive CAFs was found. In the 2 of 3 recipients who underwent HCC downstaging from above Up-to-7 to within MC by pre-LDLT therapy, recurrence of HCC was found after LDLT, and in one of these 2 cases, proliferation of α-SMA-positive CAFs was observed. On the other hand, in the one case without recurrence, proliferation of α-SMA-positive CAFs was not observed. In above Up-to-7 criteria recipients accompanied by proliferation of α-SMA-positive CAFs, the risk of HCC recurrence is very high, therefore post-operative adjuvant chemotherapy should be performed to improve survival. To improve the prognosis of recipients with HCC recurrence, appropriate anticancer therapy following recurrence is critical. We applied RFA to HCC recurrence in graft liver, and in 2 cases of recurrence with metastasis only to the lung, partial lung resection was performed. We also performed surgical resection for lymph node metastasis in the abdominal cavity. Moreover, we performed irradiation and administered molecular target drugs, and confirmed that in cases of HCC recurrence, the prognosis was improved by these intensive therapies. The proliferation of α-SMA-positive CAFs is closely related to the Up-to-7 criteria. The proliferation of α-SMA-positive CAFs, as shown in Table IV, is unrelated to pre-operative therapy or histological grade of HCC, values of tumor markers, presence of microvascular invasion, or hepatitis viruses (HBV or HCV), and appears to be a major prognostic factor in the recurrence of HCC.

From the predictors which define HCC recurrence after LT in univariate analysis, we performed a Cox-proportional multivariate analysis using three key factors such as the Up-to-7 criteria, the Tokyo criteria and proliferation of α-SMA-positive CAFs (Table V). From the results, we determined that the Up-to-7 criteria and proliferation of α-SMA-positive CAFs are both independent, most significant lyprognostic factors of LDLT for HCC.

**Figure 4:**
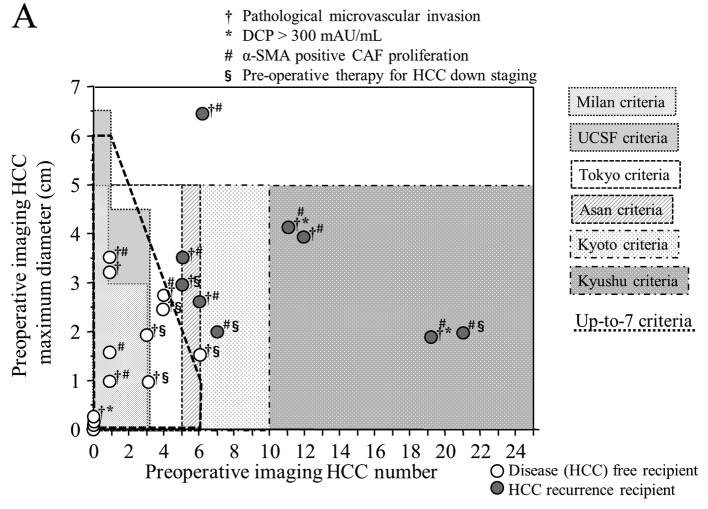

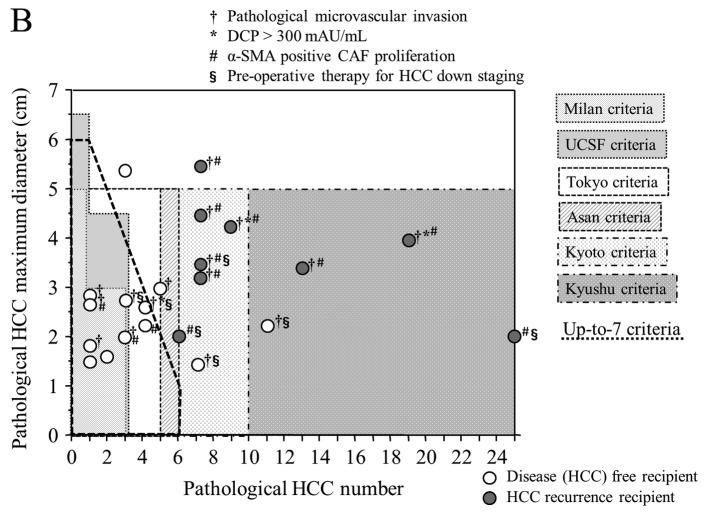
(A) Correlation between pre-operative imaging of hepatocellular carcinoma (HCC) maximum diameter and HCC number in living donor liver transplantation (LDLT) recipients (22 cases). This figure expresses the correlation between some eligibility criteria of liver transplantation (LT) for HCC with maximum tumor diameter, tumor number, microvascular invasion and proliferation of α-smooth muscle actin (α-SMA)-positive cancer-associated fibroblasts (CAFs). All 12 cases within Up-to-seven (Up-to-7) criteria survived without HCC recurrence. In above Up-to-7 criteria, only one case survived without HCC recurrence, and all other 9 cases presented HCC recurrence. (B) Correlation between pathological HCC maximum diameter and HCC number in LDLT recipients (22 cases). This figure expresses the correlation between some eligibility criteria of LT for HCC with maximum tumor diameter, tumor number, microvascular invasion and proliferation of α-SMA-positive CAFs. All 9 cases within Up-to-7 criteria survived without HCC recurrence. In above Up-to-7 criteria, all 4 cases that survived without HCC recurrence had no proliferation of α-SMA-positive CAF.

Fig. 4 shows the outcome of the present study which is the relationship between tumor number, maximum tumor diameter, microvascular invasion and proliferation of α-SMA-positive CAFs respectively for the pre-operative first imaging diagnosis prior to LDLT and pathological diagnosis. As shown in Fig. 4A based on the pre-operative first imaging diagnosis, all within Up-to-7 criteria cases survived without HCC recurrence, accounting for the largest number of cases, so these criteria appear to be the most suitable for LDLT for HCC. There were 4 cases that were diagnosed with no viable cancer lesions by pre-operative therapy for HCC such as TACL and RFA in pre-operative first imaging diagnosis, but these 4 cases had viable HCC cells. On the other hand, based on pathological diagnosis (Fig. 4B), all within Tokyo criteria recipients survived without HCC recurrence, accounting for the largest number of cases, so this would appear to be the most significant criteria from the viewpoint of recipient benefit. No proliferation of α-SMA-positive CAFs in HCC was found in the 4 recipients without post-LDLT HCC recurrence, who were diagnosed above Up-to-7 criteria by post-operative pathological diagnosis.

## Discussion

LT continues to be associated with significant morbidity and mortality despite improvements in surgical techniques and immunosuppressive regimens. Furthermore, unlike other forms of oncological surgery, LT requires a donor organ. In view of this, utility and fairness need to be considered in relation to allocation for both donor and recipient. For this reason, the application of strict eligibility criteria such as MC has evolved as an important aspect of current clinical practice. In Japan, whether or not pre-transplant therapy is performed for HCC at 3 months prior to LT, within MC, is an essential requirement for receiving DDLT, and is also an essential requirement for receiving LDLT under the health insurance. In LDLT, since a healthy donor takes a major risk, HCC recurrence must be avoided after LT in recipients. As there is no absolute curative treatment for HCC recurrence, LDLT should be performed while adhering to strict eligibility criteria so that HCC does not recur after LT. In the present study, the OS and DFS after LDLT in patients who met Up-to-7 criteria in both pre-operative evaluation and pathological evaluation were 100% although including recurrence of HCV hepatitis, we support that Up-to-7 criteria are well-established tools for assessing the prognosis of HCC. In the pre-operative first imaging diagnosis and pathological diagnosis, there were no cases which were above MC and within UCSF criteria; however, there were 2 cases which were above MC and within Up-to-7 criteria. Therefore, we did not compare Up-to-7 criteria exceeding MC and UCSF criteria but, as shown in Fig. 4, the Up-to-7 criteria broadly cover the UCSF criteria. Moreover, in view of the fact that the only patient who survived without HCC recurrence above Up-to-7 criteria was also close to within criteria, it seems most appropriate to take the Up-to-7 criteria as suitable global standard criteria for LT in HCC.

Some of the published criteria of LT for HCC do not affect the OS after LT and appear to underestimate the risk of HCC recurrence. The reason for this may be that benign/malignant borderline lesions, such as high-grade degenerative nodules (17,23–25), are counted as HCC. High-grade degenerative nodules must be clearly distinguished from HCC and a consensus has already been reached regarding this difference (17,25). If high-grade degenerative nodules are included in HCC, it detracts from the reliability of the criteria itself. Pathologically, the ideal criteria are the Tokyo criteria, but 2 cases of recurrence were found in pre-operative first imaging diagnosis, and it is difficult to conclude that Tokyo criteria would have better eligibility criteria than the Up-to-7 criteria.

### Downstaging

The downstaging refers specifically to treatment undertaken to convert a tumor with morphology beyond established LT criteria (and therefore not a candidate for LT) to a size that is within criteria and therefore enable a patient to become an LT candidate. Any assessment of the efficacy of a downstaging protocol needs not only a clear definition of which patients would be considered for downstaging, but also a clear definition of eligibility criteria that need to be met for the patient to qualify for LT. Furthermore, some protocols require a period of stability once LT criteria have been met prior to activation on waiting. Such a restriction should ensure that patients with tumors that exhibit unfavorable biology, which would be expected to translate into an increased risk of recurrence, are excluded. However, comparable post-LT outcomes in recipients who had been successfully downstaged to recipients within MC (26–28) have been demonstrated. In the present study, we performed pre-LT therapy for downstaging in 7 recipients. Pre-LT therapy consisted of 5 cases in which only TACL was performed, and 2 cases in which RFA was performed in addition to TACL. In both situations, LDLT was performed at 3 months or more after pre-LT therapy. Four of the 5 recipients above Up-to-7 criteria who underwent pre-LT therapy were judged to be within Up-to-7 criteria from above Up-to-7 criteria in the pre-operative final diagnosis, but in the pathological diagnosis, all of these cases were judged to be above Up-to-7 criteria (allowing microvascular invasion). The reasons for the discrepancy between the pre-operative final imaging diagnosis and the pathological diagnosis are that minute, residual viable cancer lesions of TACL therapy were not identified in the images, and small HCC was judged as high-grade degenerative nodules in the pre-operative diagnosis.

### α-SMA-positive CAF (myofibroblastic CAF)

Lysophostatidic acid (LPA) accelerates HCC progression by recruiting peritumoral tissue fibroblasts (PTFs) and promoting their transdifferentiation into myofibroblasts (18). Following transdifferentiation, pretumoral tissue fibroblast expressed α-SMA and enhanced proliferation, migration and invasion of HCC cells occur. In the present study, proliferation of α-SMA-positive CAFs in HCC was significantly correlated with metastasis of HCC and above Up-to-7 criteria, and was therefore significantly considered a poorer prognosis factor equivalent to Up-to-7 criteria in post-LDLT recipients with HCC. It is generally accepted that HCC originates from hepatocytes but grows and advances while fully embedded in a surrounding microenvironment with a rich content of myofibroblasts, fibroblasts, and other cell types due to the underlying cirrhosis. Liver myofibroblasts, derived from quiescent fibroblasts and hepatic stellate cells activated by the chronic injury, can be recognized by their expression of α-SMA (29,30). Myofibroblasts have been detected at the advanced edge of several different malignancies as the predominant phenotype in the CAF population (31). Although the origin of CAF remains controversial, their immunophenotypical characterization, which primarily includes α-SMA and excludes epithelial and endothelial common markers, is widely accepted (29,32,33). CAFs differ from PTFs not in terms of somatic mutations but, rather, in terms of molecular and functional differences in modulating neighboring cancer cells (34,35). However, the paracrine crosstalk between HCC and stromal fibroblasts such as CAF or pretumoral tissue fibroblast is poorly understood. Stromal myofibroblasts in HCC and matching peritumoral tissues is detected by staining with anti-α-SMA antibody (29). It was found that α-SMA-positive cells were mainly expressed within the tumor stroma (18).

We also performed an immunohistochemical study for biological markers of epithelial mesenchymal transitions (EMT) in HCC which are thought to be related to cancer invasion and metastasis (36,37) (data not shown). It is reported that downregulation of E-cadherin (36–39), weakened expression (39) or overexpression of N-cadherin (40), overexpression of β-catenin (36–38), overexpression of vimentin (41,42), overexpression of Snail (36,43), overexpression of Slug (36) and overexpression of TWIST (36,39) are poor prognosis factors for HCC. We performed immunohistochemical study of these markers. The recipients with overexpression of vimentin or Snail had significantly higher risk of HCC recurrence after LT, but it did not have as much of an impact as expression of α-SMA-positive CAFs by α-SMA immunostaining. Also, when we performed a multivariate analysis using Cox’s proportional method with Up-to-7 criteria or α-SMA-positive CAFs, and other histological factors in HCC, a clear correlation was found for Up-to-7 criteria and proliferation of α-SMA-positive CAFs. It was determined that these two factors alone were independent factors that specified prognosis or DFS after LDLT (Table V). In other words, proliferation of α-SMA-positive CAFs leads to a high risk of HCC metastasis and the prognosis is extremely poor even if LT is performed.

### Microvascular invasion of HCC

The investigated cases in the present study did not include any cases of macrovascular invasion, and since it is reported that macrovascular invasion is a significant risk factor for recurrence of HCC (2,44–46), there is no indication of LT. On the other hand, as regards microvascular invasion, it has been reported to worsen prognosis after LT for HCC (47) and there is a conflicting report that it has no effect on the prognosis (44). If we limit the discussion to within Up-to-7 criteria, it has also been reported that the presence of microvascular invasion does not define the prognosis after LT for HCC (48). As regards the MC and Up-to-7 criteria, microvascular invasion is regarded as a factor of above criteria but, in this study, microvascular invasion did not contribute to HCC recurrence after LDLT. It is more difficult to determine the presence of microvascular invasion from pre-operative imaging (49–51), and we consider microvascular invasion should be included in the within eligibility criteria of LT for HCC. The histological type such as the combined HCC type or the poorly differentiated HCC type, and the presence of intrahepatic metastasis did not contribute to HCC recurrence after LDLT.

### Pre-operative imaging diagnosis

One common methodological flaw in studies identifying clinical predictors of favorable outcome is the use of explant pathology to provide information on tumor maximum diameter and number, with the derived criteria being subsequently applied to radiological assessments of tumor burden. However, radiological staging can be limited in accuracy; indeed, review of the Eurotransplant Allocation System demonstrated a 34% accuracy of radiology in comparison to explant pathology, with tumor absent in 8.3% of patients, overstaging of the tumor in 36.2% and understaging in 10.4% (52). This is clinically significant as radiological understaging translates into inferior outcomes (53). If the precision of HCC imaging diagnosis is low, the reliability of the criteria decreases, so pre-operative imaging diagnosis must be performed accurately using multiple modalities. In order to enhance imaging diagnostic ability in HCC, the authors, in addition to dynamic MDCT and Gd-EOB-DTPA-MRI (12,13), also perform CT with angiography (CTAP and CTHA) as far as possible (14–17). We believe that by combining these tools, the ability to diagnose HCC can be enhanced to the maximum level. By performing these three tools of pre-operative imaging, the ability to diagnose benign/malignant borderline lesions and local recurrence foci after RFA or TACL therapy is also enhanced, which made it possible to obtain a pre-operative imaging diagnosis close to a pathological diagnosis. In practice, comparing the pre-operative first diagnosis and pathological diagnosis, the sensitivity of the within Up-to-7 criteria was 100%, and the specificity was 75% which is a satisfactory result. The Up-to-7 criteria which specify eligibility for LT in HCC were found to be the criteria which clearly define prognosis in the diagnostic results obtained using these three imaging diagnostic modalities. At present, in Japan, in order to receive LDLT under health insurance, the history of therapy for HCC 3 months prior to LT is not a limitation, but satisfaction of the within MC in the pre-operative final imaging diagnosis is a requirement. However, it appears there is a sufficient scientific foundation for extending this eligibility to within Up-to-7 criteria from MC.

### LDLT vs. DDLT

In general, it is said that separate consideration for LDLT for HCC is required, as the patient already has an allocated liver graft and is therefore not dependent on the donor pool. It can therefore be argued that the application of strict eligibility criteria as required with cadaveric grafts for patients with HCC is not necessary. However, in these circumstances, the risk to the donor must be incorporated into any decision, since it is clearly unethical to expose a donor to a significant risk of morbidity or mortality. Therefore, we must consider that similar criteria would apply to patients undergoing DDLT and LDLT. In fact, similar outcomes are observed in patients receiving DDLT or LDLT for HCC within Up-to-7 criteria (47). In several countries where DDLT is mainly performed for LT, the Up-to-7 criteria are accepted as the appropriate criteria (2,10,47,48,54) and it is also clear from our present study that, similarly, there must be appropriate criteria in the LDLT (48). In other words, eligibility for LT in HCC should be within the Up-to-7 criteria regardless of whether it is DDLT or LDLT.

In conclusion, the ideal eligibility criteria of LDLT for HCC is the Up-to-7 criteria and although there were some recipients in HCV hepatitis recurrence, all recipients within the criteria survived without HCC recurrence. Also, in above Up-to-7 criteria, proliferation of α-SMA-positive CAFs was found more frequently than within criteria, and this appeared to be a major factor in recurrence of HCC after LT. On the other hand, no significant correlation was found between pre-transplant treatment for HCC, histological differentiation and tumor markers with recurrence of HCC after LDLT. At present, while there is still no effective treatment for recurrence of HCC after LT and also from the viewpoint of proliferation of α-SMA-positive CAF, LT, in particular LDLT, should be limited to recipients who are within Up-to-7 criteria in pre-operative imaging diagnosis.

## Figures and Tables

**Table I:** Summary of published outcomes of liver transplantation for HCC between recipients satisfying expanded/extended eligibility criteria.

					OS (%)			DFS (%)		
						
Eligibility criteria name and definition	Authors, year (ref.)	Study design and staging method	Tumor characteristics	Cases (n)	1-year	3-year	5-year	1-year	3-year	5-year
UCSF criteria: no extrahepatic spread or macrovascular invasion. Solitary tumor with diameter ≤65 mm, or ≤3 nodules with maximum diameter ≤45 mm and total tumor diameter ≤80 mm.	Yao *et al*, 2001 (11)	Retrospective analysis. Staging: explant pathology	Within UCSF criteria and above MC	60	90		75.2			
			Above UCSF	10	50	20	-			
Up-to-7 criteria: no extrahepatic disease or microvascular invasion. Sum of number of nodules and diameter of largest nodule (cm) ≤7	Mazzoferro *et al*, 2001 (10)	Retrospective analysis. Staging: explant pathology	Within Up-to-7 criteria and above MC without microvasular invasion	283		77.7	71.2			
			Within Up-to-7 criteria and above MC with microvasular invasion	116		60.2	47.4			
			Within MC without microvascular invasion	361		81.8	76.1			
			Within MC with microvascular invasion	44		77.1	71.6			
			Above Up-to-7 criteria and without microvasular invasion	333		71.8	64			
			Above Up-to-7 criteria with microvasular invasion	338		41.7	33			
Asan criteria: no extrahepatic disease or macrovascular invasion. ≤6 nodules with maximum diameter 50 mm.	Lee *et al*, 2008 (2)	Retrospective analysis. Staging: explant pathology	Within Asan criteria and above MC	22	100	88.9	80			
			Within MC	152	86.6	79.2	76			
			Beyond Asan criteria	32	65.7	34.1	18.9			
Tokyo criteria (5–5): No extrahepatic disease or macrovascular invasion. ≤5 nodules with maximum diameter 50 mm.	Sugawara *et al*, 2007 (3)	Analysis against predefined criteria. Staging: pre-LDLT radiology (imaging).	Within Tokyo criteria	72				97	94	
			Above Tokyo criteria	6				50	50	
Kyoto criteria: no extrahepatic disease or macrovascular invasion. ≤10 nodules with maximum diameter 50 mm. PIVKA-II ≤400 mAU/ml.	Ito *et al*, 2007 (4)	Retrospective analysis. Staging: explant pathology.	Within Kyoto criteria	78			86.7			
			Above Kyoto criteria	40			34.4			
	Takada *et al,* 2007 (5)	Retrospective analysis. Staging: pre-LDLT radiology (imaging).	Within Kyoto criteria	83			87			
			Above Kyoto criteria	44			37			
Kyushu criteria: no extrahepatic disease or macrovascular invasion. Any nodules with maximum diameter 50 mm. PIVKA-II ≤300 mAU/ml.	Shirabe *et al*, 2011 (8)	Retrospective analysis. Staging: pre-LDLT radiology (imaging).	Within Kyushu criteria and above MC	48				85	80	80
			Above Kyushu criteria	6				16.7	0	0

HCC, hepatocellular carcinoma; OS, post-operative overall survival rate of recipient; DFS, post-operative HCC disease-free survival rate of recipient; Up-to-7, Up-to-seven criteria; LDLT, living donor liver transplantation; MC, Milan criteria.

**Table II:** Background characteristics of recipients who underwent LDLT for HCC according to post-LDLT with or without HCC recurrence.

Factor	All recipients (22 cases)	Recipients without post-LDLT HCC recurrence (13 cases)	Recipients with post-LDLT HCC recurrence (9 cases)
Age, years (mean ± SD)	56±4 (range 47–64)	56±4	55±3
MELD score (mean ± SD)	14±8 (range 1–30)	15±9	11±7
GV/SLV (mean ± SD)	46.3±7.0(range 36–60)	46.1±7.5	46.6±7.2
Donor age (mean ± SD)	36±12 (range 20–61)	38±13	35±12
AFP (ng/ml) (mean ± SD)	148±264	169±323	118±182
DCP (mAU/l) (mean ± SD)	183±388	85±179	323±573
Gender (female/male)	5/17	5/8	0/9
HCV/HBV	12/10	7/6	5/4
LDLT graft (Left/Right)	5/17	2/11	3/6
Post LDLT complication, n (%)			
Bile duct stenosis	6 (27)	4 (31)	2 (22)
CMV infection	9 (41)	6 (46)	3 (33)
ACR	5 (23)	2 (15)	3 (33)
Immunosuppressant			
CNI (FK/CyA)	17/5	10/3	7/2
Prednisolone, n (%)	11 (50)	8 (62)	3 (33)
MMF, n (%)	13 (59)	8 (62)	5 (56)
Child Pugh, n (%)			
A	4 (18)	1 (8)	3 (33)
B	12 (55)	8 (61)	12 (55)
C	6 (27)	4 (31)	6 (27)
UNOS TNM, n (%)			
I,II	6 (28)	6 (46)	0
IV	16 (72)	7 (54)	9 (100)^a^
UICC TNM, n (%)			
I	2 (9)	2 (15)	0
II	18 (82)	10 (77)	8 (89)
III	2 (9)	1 (8)	1 (11)
Histological grade (poorly and combined), n (%)	6 (27.2)	4 (31)	2 (22)
Microvascular invasion, n (%)	16 (73)	9 (69)	7 (78)
Bile duct invasion, n (%)	1 (5)	1 (8)	0
Intrahepatic metastasis, n (%)	11 (50)	5 (39)	6 (67)
SVR, n (%)	14 (64)	7 (54)	7 (78)
Pre LDLT treatment for HCC, n (%)	15 (68)	9 (69)	6 (67)

a P<0.05 in the comparison of the with and without post-LDLT HCC recurrence groups using χ^2^ test (analysis was considered statistically significant).

LDLT, living donor liver transplantation; HCC, hepatocellular carcinoma; GV/SLV, actual graft volume/recipient standard liver volume ratio; AFP, α-fetoprotein; DCP, des-γ-carboxyprothrombin; CMV, cytomegalovirus; ACR, acute cellular rejection; CNI, calcineurin inhibitor; HCV, hepatitis C viral hepatitis; HBV, hepatitis B viral hepatitis; MMF, mycophenolate mofetil; SVR, sustained viral responder for hepatitis C or B virus; SD, standard deviation.

**Table III:** Outcome of recipients of LDLT for HCC according to published eligibility criteria and α-SMA-positive CAF in HCC.

				OS (%)					DFS (%)				
							
Eligibility criteria name	Staging method	Classification	Cases (n)	1-year	3-year	5-year	7-year	P-value	1-year	3-year	5-year	7-year	P-value
Milan criteria (1)	Pre-operative first	Within criteria	10	10 (100%)	10 (100%)	10 (100%)	4 (100%)	<0.005	10 (100%)	10 (100%)	10 (100%)	4 (100%)	<0.001
	imaging diagnosis	Above criteria	12	10 (83%)	8 (67%)	7 (58%)	-		7 (58%)	4 (33%)	3 (25%)	-	
	Pre-operative final	Within criteria	14	13 (93%)	13 (93%)	13 (93%)	4 (77%)	<0.005	12 (86%)	12 (86%)	12 (86%)	4 (86%)	<0.001
	imaging diagnosis	Above criteria	8	7 (88%)	5 (63%)	4 (50%)	-		5 (63%)	2 (25%)	1 (13%)	0	
	Pathological diagnosis	Within criteria	7	7 (100%)	7 (100%)	7 (100%)	4 (100%)	<0.05	7 (100%)	7 (100%)	7 (100%)	4 (100%)	<0.05
		Above criteria	15	13 (87%)	11 (73%)	10 (67%)	1 (40%)		10 (67%)	7 (47%)	6 (40%)	1 (40%)	
Up-to-7 criteria (10)	Pre-operative first	Within criteria	12	12 (100%)	12 (100%)	12 (100%)	4 (100%)	<0.0005	12 (100%)	12 (100%)	12 (100%)	4 (100%)	<0.00001
	imaging diagnosis	Above criteria	10	8 (80%)	6 (60%)	5 (50%)	-		5 (50%)	2 (20%)	1 (10%)	-	
	Pre-operative final	Within criteria	15	14 (93%)	14 (93%)	14 (93%)	4 (80%)	<0.0005	13 (87%)	13 (87%)	13 (87%)	4 (87%)	<0.00005
	imaging diagnosis	Above criteria	7	6 (86%)	4 (57%)	3 (43%)	-		4 (57%)	1 (14%)	0	0	
	Pathological diagnosis	Within criteria	9	9 (100%)	9 (100%)	9 (100%)	3 (100%)	<0.01	9 (100%)	9 (100%)	9 (100%)	3 (100%)	<0.005
		Above criteria	13	11 (85%)	9 (69%)	8 (62%)	1 (35%)		6 (55%)	3 (27%)	2 (21%)	-	
Asan criteria (2)	Pre-operative first	Within criteria	14	13 (93%)	13 (93%)	13 (93%)	4 (80%)	<0.005	12 (86%)	12 (86%)	12 (86%)	4 (86%)	<0.005
	imaging diagnosis	Above criteria	8	7 (88%)	5 (63%)	4 (50%)	-		5 (63%)	2 (25%)	1 (13%)	-	
	Pre-operative final	Within criteria	15	14 (93%)	14 (93%)	14 (93%)	4 (80%)	<0.0005	13 (88%)	13 (88%)	13 (88%)	4 (88%)	<0.00005
	imaging diagnosis	Above criteria	7	6 (71%)	4 (57%)	3 (43%)	-		4 (57%)	1 (14%)	0	0	
	Pathological diagnosis	Within criteria	13	12 (92%)	12 (92%)	12 (92%)	4 (79%)	<0.05	11 (85%)	11 (85%)	11 (85%)	4 (85%)	<0.005
		Above criteria	9	8 (89%)	6 (67%)	5 (56%)	-		6 (67%)	3 (33%)	2 (22%)	-	
Tokyo criteria (5-5 rule) (3)	Pre-operative first	Within criteria	14	14 (100%)	14 (100%)	13 (93%)	4 (79%)	<0.005	13 (93%)	13 (93%)	11 (86%)	4 (86%)	<00001
	imaging diagnosis	Above criteria	8	6 (75%)	4 (50%)	3 (38%)	-		4 (50%)	1 (13%)	1 (13%)	-	
	Pre-operative final	Within criteria	16	15 (94%)	15 (94%)	15 (94%)	4 (74%)	<0.005	14 (88%)	14 (88%)	13 (81%)	4 (81%)	<0.00001
	imaging diagnosis	Above criteria	6	5 (83%)	3 (50%)	2 (33%)	-		3 (50%)	0	0	0	
	Pathological diagnosis	Within criteria	11	11 (100%)	11 (100%)	11 (100%)	4 (100%)	<0.005	11 (100%)	11 (100%)	11 (100%)	4 (100%)	<0.00001
		Above criteria	11	9 (82%)	7 (64%)	6 (55%)	-		6 (55%)	3 (27%)	2 (21%)	-	
Kyoto criteria (4,5)	Pre-operative first	Within criteria	16	15 (94%)	14 (88%)	14 (88%)	4 (69%)	**>0.05**	13 (81%)	13 (81%)	12 (75%)	4 (75%)	<0.01
	imaging diagnosis	Above criteria	6	5 (83%)	4 (67%)	3 (33%)	-		4 (67%)	1 (17%)	1 (17%)	-	
	Pre-operative final	Within criteria	17	16 (94%)	15 (88%)	15 (88%)	4 (70%)	<0.05	14 (82%)	14 (82%)	13 (76%)	4 (76%)	<0.005
	imaging diagnosis	Above criteria	5	4 (80%)	3 (60%)	2 (40%)	-		3 (60%)	0	0	0	
	Pathological diagnosis	Within criteria	16	15 (94%)	14 (88%)	14 (88%)	4 (63%)	**>0.1**	13 (81%)	12 (75%)	11 (69%)	4 (69%)	**>0.1**
		Above criteria	6	5 (83%)	4 (67%)	3 (50%)	-		4 (67%)	2 (33%)	2 (33%)	-	
Kyushu criteria (6–8)	Pre-operative first	Within criteria	18	17 (94%)	15 (83%)	14 (78%)	4 (61%)	**>0.5**	15 (83%)	13 (72%)	12 (67%)	4 (67%)	<0.05
	imaging diagnosis	Above criteria	4	3 (75%)	3 (75%)	3 (75%)	-		2 (50%)	1 (25%)	1 (25%)	-	
	Pre-operative final	Within criteria	18	17 (94%)	15 (83%)	14 (78%)	4 (61%)	**>0.5**	15 (83%)	13 (72%)	12 (67%)	4 (67%)	<0.05
	imaging diagnosis	Above criteria	4	3 (75%)	3 (75%)	3 (75%)	-		2 (50%)	1 (25%)	1 (25%)	-	
	Pathological diagnosis	Within criteria	18	17 (89%)	15 (83%)	14 (78%)	4 (61%)	**>0.5**	15 (83%)	13 (72%)	12 (67%)	4 (67%)	<0.05
		Above criteria	4	3 (25%)	3 (25%)	3 (25%)	-		2 (50%)	1 (25%)	1 (25%)	-	
α-SMA-positive CAF	Pathological diagnosis	Grade I	10	10 (100%)	10 (100%)	10 (100%)	3 (88%)	<0.0001	10 (100%)	10 (100%)	9 (90%)	3 (90%)	<0.00001
		Grade II	8	8 (100%)	7 (88%)	6 (75%)	1 (60%)		7 (88%)	4 (50%)	4 (50%)	1 (50%)	
		Grade III	4	2 (50%)	1 (25%)	1 (25%)	0		0	0	0	0	

Survival rates were estimated using the Kaplan-Meier method and compared between groups by the log-rank and generalized Wilcoxon analysis. P<0.05, analysis was considered statistically significant; P≥0.05, analysis was not considered statistically significant. LDLT, living donor liver transplantation; HCC, hepatocellular carcinoma; α-SMA, α-smooth muscle actin; CAF, cancer-associated fibroblast; OS, post-operative overall survival rate of recipient; DFS, post-operative HCC disease-free survival rate of recipient.

**Table IV:** Correlation between α-SMA-positive CAF in HCC of LDLT recipients with clinicopathological factors and published eligibility criteria.

		α-SMA-positive CAF	
		
Factor	All redipients (22 cases)	Grade I (10 cases)	Grade II, III (12 cases)
Age, years (mean ± SD)	56±4	56±3	55±4
Gender (female/male)	5/17	5/5	0/12^a^
MELD score (mean ± SD)	14±8 (range 1–30)	15±9	11±7
HCV/HBV	12/10	5/5	7/5
Child-Pugh, n (%)			
A	4 (18)	0	4 (33)^a^
B, C	12 (55)	10 (100)	8 (67)^a^
Pre-LDLT treatment for HCC, n (%)	15 (68)	7 (70)	8 (67)
AFP (ng/ml) (mean ± SD)	148±264	53±87	227±343
DCP (mAU/l) (mean ± SD)	183±388	106±202	246±508
CEA (ng/ml) (mean ± SD)	4.4±1.5	4.4±1.7	4.5±1.3
CA19-9 (U/ml) (mean ± SD)	73.4±82.6	101.7±94.7	45.1±62.7
HCC numbers (pre-LDLT first imaging diagnosis) (mean ± SD)	5.3±5.8	2.2±2.3	7.8±6.7^b^
HCC numbers (pathological diagnosis) (mean ± SD)	6.6±6.0	4.0±3.3	8.8±7.0
HCC maximum diameter (pre-LDLT first imaging diagnosis) (mean ± SD) (cm)	2.2±1.6	1.4±1.4	2.9±1.5
HCC maximum diameter (pathological diagnosis) (cm)	2.9±1.2	2.5±1.2	3.2±1.1
Sum of all HCC diameters (pre-LDLT first imaging diagnosis) (mean ± SD) (cm)	7.6±10.1	2.0±3.8	12.2±11.9^b^
Sum of all HCC diameters (pathological diagnosis) (mean ± SD) (cm)	10.3±9.4	6.6±5.9	13.4±10.8
UNOS TNM, n (%)			
I, II	6 (28)	5 (50)	1 (8)^a^
IV	16 (72)	5 (50)	11 (92)^a^
Histological grade (poorly and combined), n (%)	6 (27.2)	2 (20)	4 (33)
Microvascular invasion, n (%)	16 (73)	7 (70)	9 (75)
Intrahepatic metastasis, n (%)	11 (50)	5 (50)	6 (50)
Post-LDLT HCC recurrence, n (%)	9 (41)	1 (10)	8 (67)^a^
Recipient mortality, n (%)	8 (36)	1 (10)	7 (53)^a^
Above Milan criteria, n (%)			
Imaging	12 (55)	3 (25)	9 (75)^a^
Pathology	15 (68)	5 (33)	10 (67)
Above Up-to-7 criteria, n (%)			
Imaging	10 (46)	2 (20)	8 (80)^a^
Pathology	13 (59)	4 (31)	9 (69)
Above Asan criteria, n (%)			
Imaging	8 (36)	2 (25)	6 (75)
Pathology	9 (41)	3 (33)	6 (67)
Above Tokyo criteria, n (%)			
Imaging	8 (36)	1 (13)	7 (87)^a^
Pathology	11 (50)	3 (27)	8 (73)
Above Kyoto criteria, n (%)			
Imaging	6 (27)	1 (17)	5 (83)
Pathology	6 (27)	2 (33)	4 (67)
Above Kyushu criteria, n (%)			
Imaging	4 (18)	1 (25)	3 (75)
Pathology	4 (18)	1 (25)	3 (75)

a P<0.05 in the comparison of the with and without proliferation of α-SMA-positive CAF groups using χ^2^ tests (analysis was considered statistically significant).

b P<0.05 in the comparison of the with and without proliferation of α-SMA-positive CAF groups using the Mann-Whitney U test (analysis was considered statistically significant).

α-SMA, α-smooth muscle actin; CAF, cancer-associated fibroblast; HCC, hepatocellular carcinoma; LDLT, living donor liver transplantation; AFP, α-fetoprotein; DCP, des-γ-carboxyprothrombin; HCV, hepatitis C viral hepatitis; HBV, hepatitis B viral hepatitis; SVR, sustained viral responder for hepatitis C or B virus; SD, standard deviation; imaging, HCC in the explanted liver was evaluated by pre-operative first imaging diagnosis; pathology, HCC in the explanted liver was evaluated by post-operative pathological diagnosis.

**Table V:** Multivariate analysis for recipient OS using Cox’s proportional hazard model of statistically more significant prognostic indicators such as Up-to-7 criteria, Tokyo criteria and α-SMA-positive CAF in hepatocellular carcinoma in univariate analysis.

Predictors	Hazard ratio	

		95% CI
Up-to-7 criteria^a^	61.62	2.24–1697.97
α-SMA-positive CAF^b^	8.46	1.32–54.06
Tokyo criteria^a^	0.03	0.00–1.74

a Criteria were evaluated by pre-operative final imaging diagnosis.

b α-SMA-positive CAF was categorized for three grades.

OS, overall survival; Up-to-7, Up-to-seven; α-SMA, α-smooth muscle actin; CAF, cancer-associated fibroblast; CI, confidence interval.

## Abbreviations

AFP: α-fetoprotein
CAFs: cancer-associated fibroblasts
CTAP: computer tomography under angiography during arterial portography
CTHA: computer tomography under angiography during hepatic arteriography
DCP: des-γ-carboxyprothrombin
DDLT: deceased donor liver transplantation
HCC: hepatocellular carcinoma
DFS: disease-free survival
dynamic MDCT: dynamic multi-detectable-row computer tomography
Gd-EOB-DTPA-MRI: gadolinium ethoxybenzyl diethylenetriamine pentaacetic acid-enhanced magnetic resonance imaging
HBV: hepatitis B virus
HCV: hepatitis C virus
LC: liver cirrhosis
LDLT: living donor liver transplantation
LT: liver transplantation
OS: overall survival
RFA: radiofrequency ablation therapy
TACL: transarterial chemo-lipiodolisation
Up-to-7: Up-to-seven criteria
